# The Mesopotamia Commission Report

**Published:** 1917-07-07

**Authors:** 


### The Hospital

The Workers' Newspaper of Administrative Medicine and Institutional Life, Administration, National Insurance and Health.

No. 1622, Vol. LXII. SATURDAY, JULY 7, 1917. PRICE ONE PENNY

### THE MESOPOTAMIA COMMISSION REPORT.

Whatever other comment may be made on the Report of the Mesopotamia Commission no one has said, or will say, that it is of the whitewashing order. Dealing with a certain series of events, it cultivates within its limits both thoroughness and frankness, and neither methods nor men escape the light of day. The work thus merits recognition for its courage, and, so far as we can judge, for its impartiality. The tale is indeed a distressing one, and though in this respect it by no means stands alone in the annals of war, there exist circumstances which lend to the Report a quality of special and painful acuteness. Defeat in the field and the surrender of a British army naturally touch the national pride, and the knowledge that brave men were sent to a disastrous fate in spite of the warnings of the General in command justify a sense of impatience and of anger. These events, however, were known prior to the Commissioners' Report, and though they are marked in our memories the first emotional reaction to them has passed. It is the detailed story of the terrible and cruel sufferings of the sick and wounded soldiers that has stirred to its depths the heart and conscience of the nation and has sharpened the demand for the identification and punishment of those who are responsible for such a record. The position is further emphasised by contrast with the generally accepted account of the success of the Medical Services on the Western Front, for this makes clear to demonstration the conclusion that by efficient organisation the horrors of the Eastern campaign could have been avoided. A further note of anger is added by the sustained and repeated "reticence" of those occupying high places in the military hierarchy, and for this the general public finds a more emphatic term. The harm in this respect is not limited to the particular incident now in question. On the contrary, it extends to official methods generally, and tends towards that supreme disaster in times of national crisis—namely, the suspicion on the part of the people that they are being betrayed and deceived. The events themselves are bad enough in all conscience, and the setting of them in a fair frame of official falsehood adds to the gloom of the picture. For neither the one nor the other is forgiveness possible.

The one bright spot in the record is provided by the courage and resolution of the officer who did not hesitate to risk his professional position and prospects in the cause of frankness and of truth. What Lieutenant-Colonel Carter's stand meant for him is fairly well indicated by the official threat to place him under arrest as an "interfering faddist." With moral courage of the highest order he refused to aid in the suppression of the truth, even though his refusal involved the apparent certainty of the official censure which stands as a permanent handicap on an officer's career. The whole nation has read his account of the actual condition of the wounded as these were removed by river to Basra, and for this and for the high standard of duty of which it is the index he is rightly the recipient of general respect and gratitude. Such service must not go unrewarded. To it we owe very largely both the discovery of the truth and what goes with such discovery—the opportunity of prevention for the future. Every organisation makes a large claim to mutual loyalty on the part of those who compose it, and this claim has its measure of justification. But there is a deeper and a larger loyalty, and it is because he has seen and responded to this that Lieutenant-Colonel Carter, amidst the fall of reputations, is set on a high place in the public esteem.

We do not propose to enter any judgment on the conduct of those who have come under the censure of the Commissioners. The trial has been conducted by a competent tribunal, and, we believe, in an impartial fashion. Those concerned have had an opportunity of putting in their explanations and defences. Hence, whatever may be said in mitigation of punishment, we cannot see how it is possible to re-try the issues in the columns of newspapers. We readily believe that certain individuals have been largely the victims of circumstance, but it is to overcome circumstance that men are elected to high command and opportunity. Similarly, it is a fair thing to say that some of the verdicts of the Commission are the fruit of the wisdom that cometh after the event, but here again foresight and sound judgment are qualities which are denanded from those who undertake the leadership f nations. To fail in these respects is not criminal, but the failure shows that the persons concerned were below the measure of powers reasonably expected from them. The tests are severe, and are applied in an unrelenting field. For the justification of these conditions is that without them national safety is deprived of its shield.

The true lesson to be learned from all this bitter experience of medical failure in the field is not to be found in the immediate events or in the doings of individuals suddenly thrust into the searching light of publicity. It is to be noted rather in a story which goes back into the easy or slothful days of peace. The evidence is plain that for years before the war the military authorities were plainly warned of the utter inadequacy of the medical arrangements connected with the Army in India. Sir Alfred Keogh says without qualification that they have "for years and years been most disgraceful." The financial authorities steadily refused to listen to the medical demands, and cultivated economy at the expense of efficiency. When the great test came there was the inevitable collapse, and the medical officers for the time being are involved in the disaster. On them has been laid a great share of the blame, but the real responsibility lies much further back, and those who refused the means of adequate equipment must bear the weight of it.

